# A Case of Cutaneous Fungal Infection Following the Administration of Dupilumab

**DOI:** 10.7759/cureus.81349

**Published:** 2025-03-28

**Authors:** Shinichi Tanemura, Yoshihito Mima

**Affiliations:** 1 Department of Dermatology, Kanto Central Hospital, Tokyo, JPN; 2 Department of Dermatology, Tokyo Metropolitan Police Hospital, Tokyo, JPN

**Keywords:** atopic dermatits, dupilumab, fungus, microbiome, th-17, th2

## Abstract

Atopic dermatitis (AD) is a chronic inflammatory skin condition with a multifactorial etiology. Herein, we report a case of a patient with AD undergoing long-term topical treatments who developed a dermatophyte infection following the administration of dupilumab. Dupilumab is known to enhance skin barrier function and induce changes in the skin microbiome. Notably, head and neck dermatitis caused by the overgrowth of *Malassezia* species due to dupilumab has been widely discussed. This phenomenon is thought to result from the suppression of T helper (Th)2 cytokines by dupilumab, leading to a decrease in the proportion of *Staphylococcus aureus* and a relative increase in fungal populations. Additionally, feedback activation of Th17 cytokines may trigger excessive inflammation against fungi, contributing to fungal infections. IL-13 plays critical roles in fungal colony formation, and tralokinumab, an IL-13 inhibitor, has shown potential efficacy in treating this head and neck dermatitis. While the relationship between microbiome changes and biologics like lebrikizumab and nemolizumab remains unexplored, investigating the differential effects of these therapies on the cutaneous microbiome could provide deeper insights into not only the unique characteristics of each biologic agent but also the roles of Th2 cytokines such as IL-4, IL-13, and IL-31 in the pathophysiology of AD. The present case underscores the importance of the comprehensive therapeutic approach for AD that accounts for microbiome dynamics and adapts to evolving skin changes throughout the course of treatment.

## Introduction

Atopic dermatitis (AD) is a chronic inflammatory skin disease characterized by repeated exacerbations and remissions of inflammation. AD shows diverse clinical manifestations and multifactorial backgrounds [1,2]. The key factors in the pathogenesis of AD include elevated T helper (Th)2-mediated cytokines and filaggrin gene mutations [3-6]. The cutaneous microbiome regulates the skin's immune system and subsequently contributes to the maintenance of skin homeostasis [7]. Therefore, fluctuations in the skin microbiome, particularly involving *Staphylococcus aureus *(*S. aureus*), are strongly associated with the development of AD [7].

In the skin of patients with AD, the colony formation of *S. aureus* is notable [6]. The toxins produced by these bacteria nonspecifically activate T cells and enhance the production of Th2 cytokines [6]. This activated inflammation leads to decreased microbiome diversity and immune destabilization in the skin, possibly associated with the onset and exacerbation of AD [4-7].

Dupilumab is a fully human monoclonal antibody that targets the interleukin (IL)-4 receptor alpha subunit (IL-4Rα), a component shared by the receptors for IL-4 and IL-13 [8]. IL-4 and IL-13 are involved in the Th2 immune response, associated with the pathogenesis of AD [8]. By binding to IL-4Rα, dupilumab inhibits the signaling pathways of IL-4 and IL-13. Therefore, dupilumab has been proven effective for AD [8]. Dupilumab is used to treat moderate to severe atopic dermatitis (AD) that has not improved adequately with existing treatments [4].

 IL-4 and IL-13 can inhibit the differentiation and function of Th17 cells, potentially enhancing the Th17 response and subsequently developing unexpected dermatitis such as fungal infections or psoriasiform dermatitis [9,10]. Additionally, dupilumab can induce changes in the skin microbiome and increase the proportion of filamentous fungi and *Malassezia* species, while dupilumab improves skin barrier function and reduces the proportion of *S. aureus* [2].

For example, the impact of dupilumab on the microbiome includes dupilumab-associated head and neck dermatitis, accompanied by an increasing population of *Malassezia* species [11]. While the skin barrier function overall the body improves after the administration of dupilumab, the onset of newly developing dermatitis in the localized region, such as the face and neck, can occur. Moreover, the existing skin barrier defects in the local regions may facilitate the overgrowth of *Malassezia* species [11]. This suggests the possibility that dupilumab may influence the microbiome and the increasing risks of fungal infections. Thus, a detailed discussion on the relationship between microbiome changes and fungal infections within dupilumab therapy is needed. As mentioned above, the suppression of Th2 cytokines by dupilumab may alter the skin's immune balance and microbiome composition, potentially resulting in a higher probability of fungal infections development [11,12].

Herein, we report a case of a patient with AD who developed extensive erythema due to filamentous fungal infection following the initiation of dupilumab. This case highlights new insights into the connection between the changes in the microbiome and the emergence of fungal infections during AD treatment.

## Case presentation

**Figure 1:**
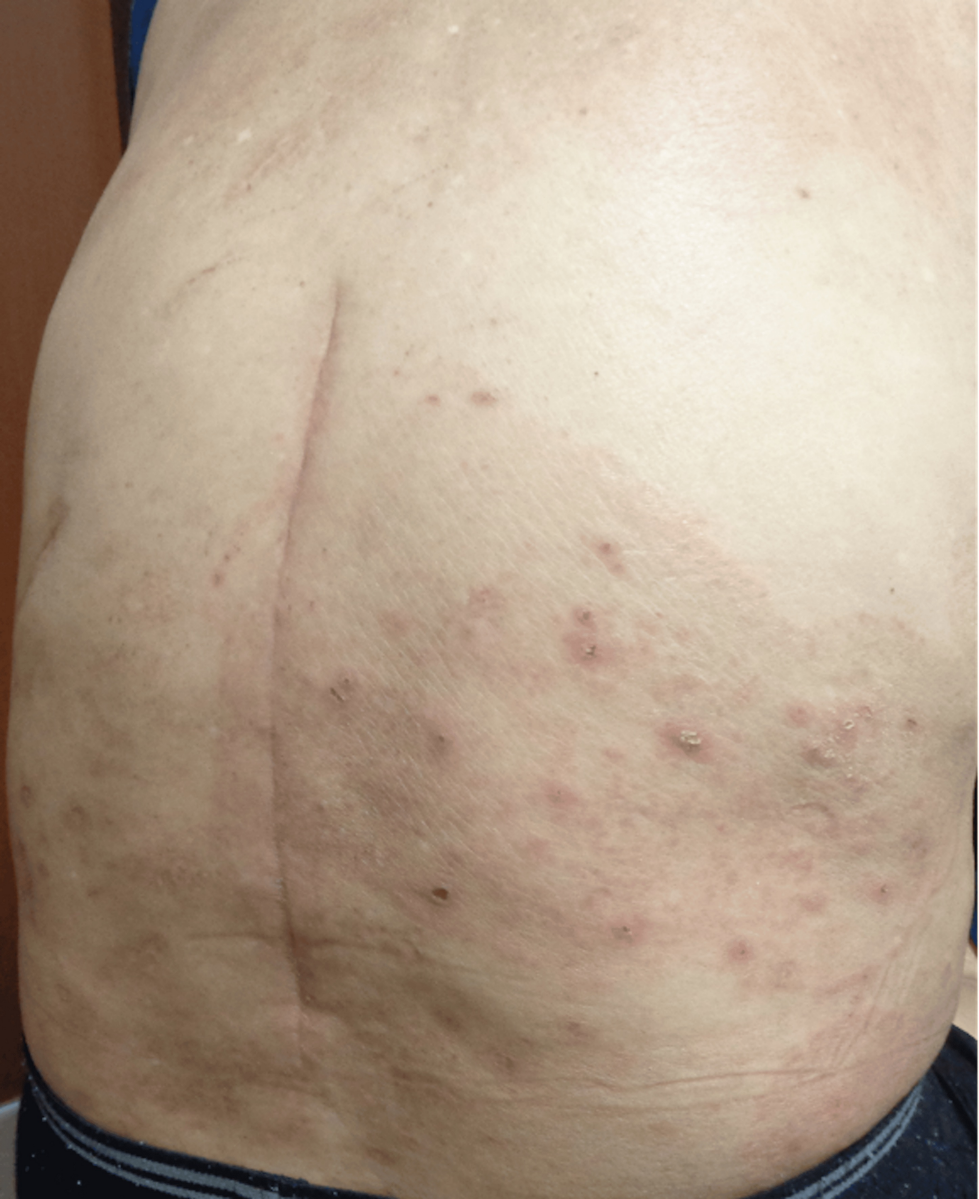
An annular erythema with central clearing was noted on the lumbar area.

**Figure 2:**
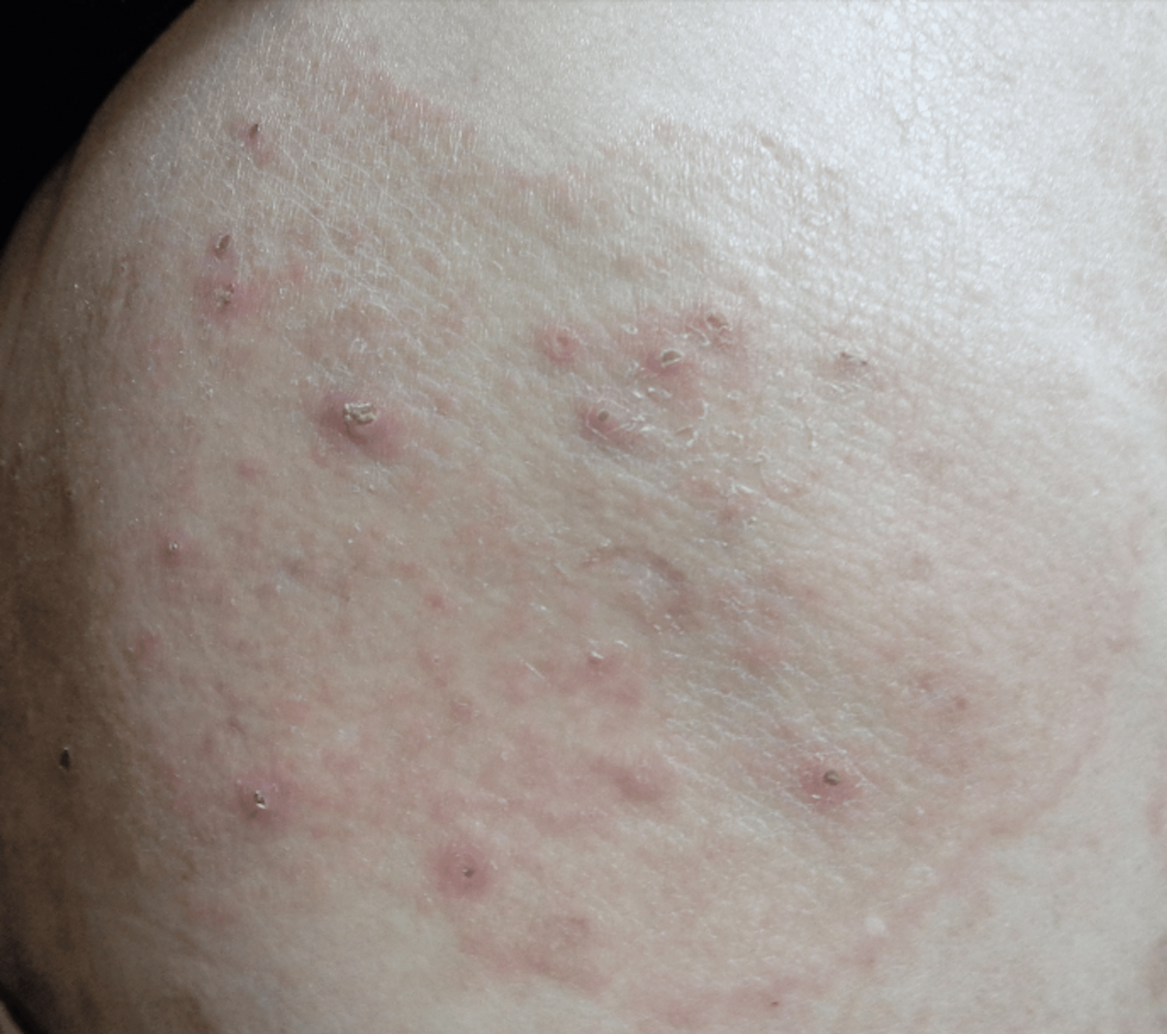
Red nodules with crusts were observed within the annular lesion, and the periphery was elevated like a ridge.

A 68-year-old man who had been clinically diagnosed with AD for more than 10 years was managed with topical steroids and oral antihistamines. He also has well-controlled hypertension and diabetes. Physical examination revealed eczematous lesions with excoriation marks and numerous prurigo nodules, refractory to topical steroids and oral antihistamines. Therefore, we decided to initiate dupilumab to improve skin symptoms and itching. Subsequently, skin symptoms and itching showed marked improvement following the administration of dupilumab. However, after the fifth administration of dupilumab, an itching erythema with central healing tendency developed on his right lumbar region (Figures 1, 2). 

**Figure 3:**
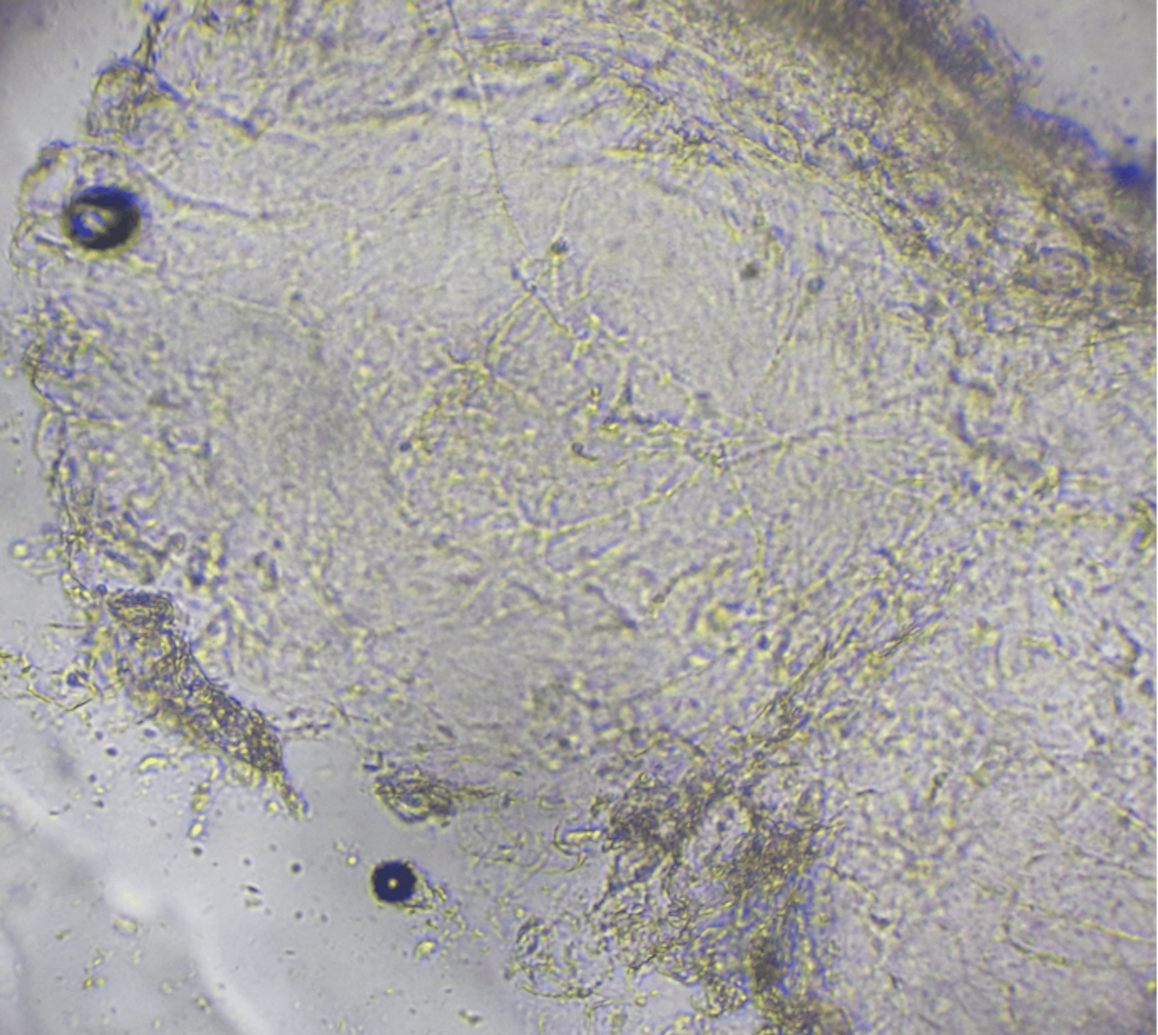
Fungal tests revealed filamentous fungi.

The fungal tests of the annular lesions were positive (Figure 3), and no bacteria were detected in the bacterial culture test of the annular lesion. Therefore, the patient was diagnosed with a fungal infection.

After that, topical anti-fungal therapy was initiated, along with topical corticosteroid therapy. The frequency of topical corticosteroid application was reduced from once daily to twice weekly. Two months later, the eruptions completely resolved.

## Discussion

In the present case, the erythema with central healing tendency appeared on the right lumbar region after the fifth administration of dupilumab, though the pruritus and skin symptoms of AD markedly improved. Within the lesion, red nodules with crusts were observed, and the periphery was elevated like a ridge. The positive result of fungal tests indicated that the rash was caused by filamentous fungal infection. Dupilumab improves cutaneous symptoms in patients with AD by inhibiting the signaling pathways of IL-4 and IL-13, thereby suppressing Th2 immune responses [1]. The administration of dupilumab has been reported that the improvement of the skin barrier function and the inflammation in atopic dermatitis contributes to the difficulty of *S. aureus* to settle in the skin and to the reduction of the rate of *S. aureus* [2]. On the other hand, an increasing portion of filamentous fungi in the skin has been noted during dupilumab treatment [2]. IL-4 and IL-13 can inhibit the differentiation and function of Th17 cells [12,13]. The inhibition of these cytokines accompanied by dupilumab addiction may lift this inhibition, potentially enhancing Th17 responses [12]. Th17 activation by dupilumab may induce hypersensitivity reactions and inflammation against fungal infections [13]. Thus, dulipumab may also contribute to fungal infection through Th17 immune response in the present case.

In this case, dupilumab treatment improved skin symptoms in AD with increased T helper 2 (Th2)-associated cytokines and filaggrin gene mutation. However, the prolonged and excessive use of topical corticosteroids, along with changes in the microbiome, is considered to contribute to the increasing proportion of filamentous fungi, ultimately leading to the onset of fungal infection [2].

The patient was a long-term user of topical corticosteroids with a high risk of fungal infection [14], but no fungal infection occurred prior to the initiation of dupilumab. Therefore, we consider that dupilumab may change the microbiome in the skin, and then lead to the onset of fungal infection. Sudden discontinuation of topical corticosteroids in the settings of fungal infections can even further exacerbate skin lesions due to immune reconstitution [15]. Therefore, we opted to continue topical corticosteroid application twice a week in addition to using the anti-fungal ointment.

Following the introduction of dupilumab as a biologic treatment for AD, three additional biologics (nemolizumab, tralokinumab, and lebrikizumab) have emerged [16-18]. Nemolizumab specifically binds to IL-31 receptor A (IL-31RA), blocking IL-31 signaling and thereby reducing itch and inflammation of AD [17]. Tralokinumab directly binds to IL-13, preventing its interaction with both IL-13Rα1 and IL-13Rα2 and broadly inhibiting IL-13-dependent signaling pathways [16]. In contrast, lebrikizumab binds to IL-13Rα1 with high affinity, selectively blocking its interaction with IL-4Rα and thus inhibiting IL-13 signaling [18]. All three biologics, like dupilumab, target Th2 cytokines (IL-4, IL-13, and IL-31) and have demonstrated efficacy in the treatment of AD through clinical trials [16-18].

Tralokinumab has been suggested as a potential treatment for dupilumab-associated head and neck dermatitis due to the increasing colonization of *Malassezia* species [19]. This may be attributed to the fact that, unlike dupilumab, tralokinumab does not inhibit IL-4 signaling pathways, making Th17 hyperactivation less likely and thereby reducing hyperactivated inflammation toward fungal infections [19]. Additionally, IL-13 plays crucial roles in maintaining the homeostasis of *Malassezia* colony formation. By broadly inhibiting IL-13, tralokinumab may suppress *Malassezia* colonization, further contributing to its effectiveness against head and neck dermatitis associated with dupilumab [12,19]. These mechanisms suggest that tralokinumab could be a viable option for addressing this specific adverse effect of dupilumab [12,19].

Currently, the relationship between the cutaneous microbiome and nemolizumab or lebrikizumab remains unexplored. While both tralokinumab and lebrikizumab inhibit IL-13, their mechanisms differ: tralokinumab blocks both IL-13Rα1 and IL-13Rα2, whereas lebrikizumab specifically targets IL-13Rα1 [16,18]. These differences in their mechanisms may lead to distinct effects on the fungal and bacterial microbiomes. Further investigation into these differences in microbiome changes could also provide insights into the specific roles of IL-13Rα1 and IL-13Rα2, which remain incompletely understood.

To advance our understanding of the roles of IL-4, IL-13, and IL-31 in AD, it is essential to explore the impact of tralokinumab, lebrikizumab, and nemolizumab on the skin microbiome. Such studies may reveal critical distinctions in how these biologics interact with the microbiome and contribute to the broader understanding of AD pathogenesis and treatment.

When new erythema or skin eruptions develop during the treatment of AD, it is essential to consider not only the exacerbation of AD but also the possibility of fungal infections. Careful skin observation is critical in AD management, especially given the potential changes in the microbiome [2]. Furthermore, providing patients with proper guidance on the frequency and method of topical treatments in daily life is crucial for preventing the recurrence of fungal infections. While the relationship between dupilumab and skin microbiome has been actively discussed, there has been minimal exploration of the microbiome's interaction with other biologic agents for AD, such as tralokinumab, lebrikizumab, and nemolizumab. Future research in this area could enhance our understanding of the unique characteristics of each biologic agent in AD treatment. This case underscores the necessity of adopting a comprehensive treatment strategy for AD that considers microbiome changes in the management of AD.

## Conclusions

We report a case of a patient with AD who developed a dermatophyte infection after the initiation of dupilumab. When erythema recurs during AD treatment, it is necessary to consider the possibility of fungal infections due to changes in the microbiome. The increase in the relative proportion of fungi and the activation of Th17 cytokines due to the suppression of Th2 immune response following dupilumab administration have been suggested as potential contributors to the development of fungal infections. In AD treatment strategies, it is important to keep microbiome alterations in mind and provide appropriate guidance on the use of topical therapies and biological agents. Further accumulation of cases and research on the role of the microbiome in AD treatment is needed.
